# Optimized microwave assisted extraction (MAE) of alkaloids and polyphenols from Berberis roots using multiple-component analysis

**DOI:** 10.1038/s41598-020-57585-8

**Published:** 2020-01-22

**Authors:** Tarun Belwal, Aseesh Pandey, Indra D. Bhatt, Ranbeer S. Rawal

**Affiliations:** 1 G.B. Pant National Institute of Himalayan Environment and Sustainable Development, Kosi Katarmal, Almora, 263643 Uttarakhand India; 2 G.B. Pant National Institute of Himalayan Environment and Sustainable Development, Sikkim Regional Centre, Pangthang, Gangtok, 737101 Sikkim India

**Keywords:** Chromatography, Secondary metabolism

## Abstract

*Berberis*, one of the major sources of berberine and polyphenols, is widely accepted genus for its medicinal properties. The inclusion of these phytochemicals in different health formulations has widened its scope in pharmaceuticals and nutraceuticals. In the present study, multi-component analysis (MCA) has been used to extract these nutraceutical compounds from *Berberis jaeschkeana* roots under microwave-assisted extraction (MAE) conditions. To determine the optimum extraction condition, different factors, including, microwave power, sample to solvent ratio, irradiation time, solvent pH and solvent concentration were tested under 42 experiments. The MCA includes, Plackett-Burmen and Central Composite Design and analyzes model fitness, regression coefficient (β), analysis of variance (ANOVA) and 3D response curve. The results showed significant model fitness and involvement of linear, quadratic and interactive effect of different factors. Under optimized MAE condition, [i.e. 1 g of sample extracted through 70 mL of a solution (100% methanol pH 2.0), provided microwave power of 598 W for 2 min of irradiation time], the berberine and palmatine contents were recorded as 46.38 mg g^−1^ and 20.54 mg g^−1^ respectively. Under optimized condition, the yield of alkaloids were found closer to the models’ predicted value. Similarly, total phenolic content and antioxidant activities were also found closer to the models’ predicted value. To test the suitability of the optimized MAE condition for other species i.e., *Berberis asiatica*, extraction of alkaloids and polyphenolics was conducted and recorded higher yield to the previous records. Moreover, under optimum extraction condition, six and seven polyphenolic compounds from *B. jaeschkeana* and *B. asiatica* were quantified respectively. The proposed MAE optimization design using MCA contributes towards faster and greener extraction of alkaloids and polyphenolics with higher yield. Moreover these greener approaches could sustainably utilize species during extract preparation and harnessing its nutraceutical and pharmaceutical potential. This study design could also be replicated on other valuable species or compounds for effective extraction of nutraceutical components and sustainable utilization of natural products.

## Introduction

Berberine and palmatine alkaloids are chemically complex compounds of protoberberine benzylisoquinoline class and largely found in the *Coptis, Berberis, Mahonia, Hydrastis, Xathorhiza, Phellodendron, Tinospora, Argemone* and *Eschscholzia* plant genus^1^. Among others, *Berberis* spp. are distributed worldwide with over 500 species^2^ and known to contain berberine, and palmatine as major alkaloids^3,4^.

Alkaloids, especially berberine from *Berberis* species, are well explored for its anti-diabetic^5^, hepato-protectant^6^, anti-arthritic^7^, anti-oxidants^8^, anti-microbial^9^, neuro-protective^10^ and hypo-lipidemic^11^ activity. The berberine is also reported for its antioxidant activity by activating antioxidant enzymes^12^ and scavenging reactive oxygen species (ROS)^13^. This is also known to lower the risk of cardiovascular associated diseases^14^. Thanks to these potential health promoting activities of berberine, a vast number of pharmaceutical and nutraceutical products are available in the present market. For instance, ‘Armolipid Plus’ which contain berberine is used to lower the high cholesterol/lipid level and is largely marketed in Europe^15^ and most frequently employed in Italy^16^. Similarly, ‘Zyflamend Softgel Capsule’, which acts as antioxidant and anti-inflammatory dietary supplement to manage osteoarthritis and rheumatoid arthritis also contain berberine^17^.

Polyphenols are also reported from *Berberis* species, mainly from its fruit part^18–23^. Polyphenols are widely searched compounds and found effective against a number of disease conditions, such as, cancer, down syndrome, diabetes, Alzheimer, Parkinson and inflammation^24–28^.

To obtain the desired secondary metabolites and/or extract as a whole, plant materials undergoes various processes, including drying, extraction, separation and purification^21,29^. These bioprocessing conditions have direct effect on the extraction yield and extract quality, thus determine the final product cost. Traditionally, *Berberis* root extract was prepared by boiling, decoction and infusion, while, maceration and microwave assisted extraction (MAE) techniques are used during laboratory experiments^29,30^. Reports revealed that various compounds of nutraceutical and pharmaceutical interest have been extracted by MAE method^21,31–33^. The MAE is reported as a green extraction method, as it provides both faster extraction and lesser or no solvent consumption^34,35^. The excess heat and pressure developed during the process increases mass transfer and helps in extracting plant components in lesser time with increased yield as compared to conventional extraction methods^31,36–41^. Excessive heat during MAE was also reported to degrade the heat sensitive compounds^32,33,35,42^, thus the processing conditions (i.e., microwave power, irradiation time, type of solvents and composition) needs to be optimized for obtaining better quality of extract and compounds.

To determine the optimum processing conditions, various efforts have been made. As such, single factor analysis (univariate) determines only the effect of a single variable at a time over the responses and neglects the interactive effect between variables, thus leads to a larger number of experiments with futile results. On the other hand, a multiple-component analysis (MCA) not only determines the individual effect but also the interactive effects of variables^41^. Response surface methodology (RSM) is one such MCA, which needs lesser experimental runs and thus could be a choice for optimization of process conditions^43,44^. To date, the extraction of berberine and other alkaloids have successfully been optimized from stem part of *Berberis amurensis*^30^ and rhizome of *Coptis chinensis*^45^ using RSM. Although, the root part of *Berberis* spp. is reported to contain the higher amount of alkaloids^46–48^, however the optimum extraction condition has not yet been developed under advanced extraction technique. Thus, this study was designed to apply MCA, including Plackett-Burman design (PBD) and central composite design (CCD) for optimizing MAE conditions for alkaloids and phenolics from *B. jaeschkeana* C.K. Schneid roots. Also, the optimized MAE extraction conditions were tested for their suitability/reliability in *Berberis asiatica* roots.

## Material and Methods

### Plant material

Roots of two Berberis species, i.e., *B. jaeschkeana* and *B. asiatica* were collected from 10 different plants of each species growing in their natural populations, i.e. from Tungnath, Garhwal (3300 to 3500 m asl) and Kosi, Almora, Kumaun region (1200 m asl) of state Uttarakhand, India, respectively. The samples were washed properly and dried under shade. Roots were cut into small pieces and grounded in a hammer mill (Model-WGM 197, UTS Sales, Delhi, India) up to <85 micron particle size. Root powder was stored at 4 °C in a refrigerator and extracts were prepared within 2 days of grinding.

### Chemicals and reagents

Sodium bicarbonate, potassium persulphate, sodium acetate, acetic acid and hydrochloric acid were purchased from Qualigens (Mumbai, India), and 2,2-azinobis (3-ethylbenzothiazoline-6-sulphonic acid) (ABTS), and methanol from Merk (Darmstadt, Germany). Sodium chloride was procured from HiMedia Laboratories (Mumbai, India). 2,2-Diphenyl-1-picryhydrazyl (DPPH), ascorbic acid, and all polyphenolic standards (i.e. rutin hydrate, phloridzin dihydrate, *p*-coumaric acid, (+)-catechin hydrate, gallic acid, quercetin dihydrate, 3-hydroxybenzoicacid, 4-hydroxybezoicacid, ellagic acid, vanillic acid, caffeic acid, *m*-coumaric acid, ferulic acid, *trans*-cinnamic acid and chlorogenic acid) and alkaloids (i.e. berberine and palmatine) were procured from Sigma Aldrich (St. Louis, Missouri, United States). All chemicals were of analytical or HPLC grade and the solutions were prepared with methanol and lab ultrapure water (Rions India Lab Water Systems, India).

### Microwave assisted extraction and phytochemical analysis

#### Microwave assisted extraction (MAE)

MAE was carried out using multiwave-3000 microwave reaction system (Anton-Paar, Germany, GmbH) consisted of 8 closed extraction vessels equipped with infrared temperature sensor, vessel mark sensor, controllers and a magnetic stirrer at the base. For extraction, 1 g of the powdered root sample was dissolved in different volumes and concentrations of methanol at different pH values (Supplementary Tables 1, 2). The mixture was placed in closed vessel extracting chamber inside the microwave reaction system. Microwave power at different levels and time periods (Supplementary Tables 1, 2) was applied according to the model design and the filtered extract was stored at −20 °C. All dependent variables (responses) were measured within two weeks from the storage time.

#### Analysis of total phenols (TP)

The TP was measured by Folin-Ciocalteau’s colorimetric method^49^. The quantification of TP content was done using a gallic acid standard curve and estimated as mg gallic acid equivalent/g dry sample (mg GAE/g dw). Briefly, 0.5 mL of Folin–ciocalteu solution was added to diluted 5 mL of extract. Thereafter, sodium bicarbonate (Na_2_CO_3_; 7% w/v) was added to the mixture. After proper mixing, the mixture was kept for 90 minutes at room temperature in dark. The absorbance of resultant blue color was measured at 765 nm under spectrophotometer (Hitachi U-2001, Japan).

#### *In vitro* antioxidant activity

The *in vitro* antioxidant activity of root extract was analyzed following Belwal *et al*. (2016). A total of three *in vitro* antioxidant assays viz. 2, 2-Diphenyl-1-picryhydrazyl (DPPH), 2, 2-azinobis-3-ethylbenzthiazoline-6-sulphonic acid (ABTS) and Ferric reducing antioxidant power (FRAP) were performed. Standard curve for all assays was prepared by ascorbic acid and results expressed in mM ascorbic acid equivalent/g dry sample (mM AAE /g dw).

### Determination of alkaloids and polyphenolic compounds

#### Alkaloids

Alkaloids (berberine and palmatine) were analyzed using high performance liquid chromatography (HPLC) (LC-10AT, Shimadzu Liquid Chromatography, Japan) equipped with the binary pump and diode-array detection (DAD-MZOA) unit^50^. Briefly, 10 µL of the (1 mg of dry extract dissolved in 1 mL of methanol) extract was injected and separated using C18 reverse phase column (250 mm × 4.6 mm i.d., 5 µm, Purosphere, Merck, Darmstadt, Germany) maintained at 25 ± 1 °C. The mobile phase comprises acetonitrile (A) and 0.13% potassium dihydrogen phosphate of pH 2.5 (B) at 50:50 ratio and flow at 1.0 mL/min for 20 min of total run time. Depending on the lambda max, detector wavelength was set at 345 nm and berberine and palmatine content was recorded as mg 100 g^−1^ dry weight. All experiments were conducted in triplicate (n = 3).

#### Polyphenolics

Moreover, polyphenolics were analyzed as per the method^20,51^, with minor modifications. Briefly, 10 µL of the (1 mg of dry extract dissolved in 1 mL of methanol) extract was injected and separated using C18 reverse phase column (250 mm × 4.6 mm i.d., 5 µm, Purosphere, Merck, Darmstadt, Germany) maintained at 25 ± 1 °C. The mobile phase consisted of a mixture of methanol and 0.1% (v/v) ortho-phosphoric acid at the ratio of 40:60 (v/v) and the flow rate was maintained at 0.8 mL/min for total run time of 40 min. The DAD wavelengths were selected from 254 to 330 nm. A total of fifteen polyphenolic standards were used and the final concentration of compounds was determined as mg 100 g^−1^ dry weight. All experiments were conducted in triplicate (n = 3).

### Multiple-component analysis (MCA)

The experiments were designed for determining optimum MAE conditions for maximizing alkaloid and polyphenolic contents. Factors such as microwave power, sample to solvent ratio, solvent pH, solvent concentration and irradiation time were selected as independent variables for alkaloids and polyphenolic extraction under MAE (Supplementary Tables 1 and 2).

For MCA, a systematic approach was followed. The independent variables were first screened based on the significant effect over the responses using Plackett-Burman design (PBD). PBD only measures linear effect of individual factors, hence PBD was conducted to determine the significant effect of microwave power, irradiation time, sample to solvent ratio, solvent pH and concentration at two levels over the response variables (i.e., berberine, palamatine and TP content) (Supplementary Table 1). The significant factors were then tested using central composite design (CCD) to determine the linear, quadratic and interactive effects over the responses (i.e., berberine, palamatine, TP content and antioxidant activity) (Supplementary Table 2). The second order polynomial equation was applied to determine the effects as:

1 $$Y={\beta }_{0}+\mathop{\sum }\limits_{i=1}^{k}\beta iXi+\mathop{\sum }\limits_{i=1}^{k}\beta iiXi2+\mathop{\sum }\limits_{i=1}^{k}\mathop{\sum }\limits_{j=i=1}^{k-1}\beta ijXiXj$$

where, *Y* is the response variable, *Xi* and *Xj* are the independent variables, and *k* is the number of tested factors (*k* = 4). The regression coefficient is defined as *β*_0_ for intercept, *βi* for linear, *βii* for quadratic and *βij* for the cross-product term. 3D graphs were generated using regression coefficient and analysis of variance (ANOVA) was conducted to find the significant (p < 0.05) effect of the model terms. For model fitness, model *F-value*, lack of fitness and coefficient of determination (R^2^) were estimated for each response variable. For multiple-component experimental design, Design Expert, v. 10.0 software (State-Ease, Inc., MN, USA) was used.

### Optimum MAE condition and Validation of the model

Keeping all the responses as maximum and factors level within the range, the optimal MAE condition for alkaloids, polyphenols and antioxidant activity were determined and validated. The optimal conditions were generated by the model based on the response value and desirability was used to select the best optimal MAE condition. The experiment was further conducted (in triplicate) on the selected optimum extraction condition and coefficient of variation (CV) was determined to validate the model. To test the reliability of the optimized MAE condition, root samples of *Berberis asiatica* were also tested for alkaloids and polyphenolic antioxidants extraction.

### Statistical analysis

The obtained results were subjected to analysis of variance (ANOVA) and all the experiments were conducted in triplicates. The statistical software used was SPSS V.17.0 (IBM Corporation).

## Results and Discussion

### Screening factors (Plackett-Burman Design)

#### Microwave power

Microwave power was used at two levels (100 and 300 W) for PBD and showed a significant variation in berberine, palmatine and TP content (Supplementary Table 1). All the responses increased with increasing microwave power, which might be due to increase in mass transfer by microwave^36,37^. Meanwhile, the excess heat also leads to degradation of compounds^35,42^, which depends on the irradiation time and physiochemical properties of compounds and has not been seen during the current study, even at higher microwave power.

#### Irradiation time

Irradiation time did not show any significant difference in the responses (Supplementary Table 1), however, lower irradiation time was found equally effective as higher. Considering the energy loss and possibility of degradation of compounds at higher irradiation time, it was kept at a lower level for CCD model.

#### Sample to solvent ratio

A positive non-significant variation in berberine content was recorded with varying sample to solvent ratio. However, for palmatine and TP content, a positive significant effect of sample to solvent ratio has been observed (Supplementary Table 1), thus can be tested at higher levels in CCD model.

#### Solvent pH

A significant effect of solvent pH was recorded for berberine and palmatine content (Supplementary Table 1). The pH levels for PBD model were set at acidic (pH 2.5) and near neutral (pH 6). Increasing the solvent pH from acidic to near neutral, a significant increase in the degradation of berberine and palmatine was observed. Thus, for CCD model the solvent pH levels were kept at lower value.

#### Solvent concentration

For MAE condition, methanol was selected as solvent of choice because of the fact that it has higher solubility of compounds of interest and also having higher dissipation factor (tan δ), which provides greater microwave heat absorption and transfer by the solvent under MAE^31,37^. As such, increasing methanol concentration from 20 to 80%, berberine and palmatine concentration increases significantly (Supplementary Table 1), thus further tested at higher levels in CCD model.

Overall, among different tested independent variables under PBD, microwave power, sample to solvent ratio, solvent pH and concentration showed significant variations in the responses. Also, the model *F-value* for berberine, palmatine and TP was found significantly fit with good coefficient of determination (R^2^) (Supplementary Table 1).

### Multiple-component optimization (central composite design)

#### Fitting the model

The CCD showed a significant (p < 0.05) model fitness (Table 1). The coefficient of determination (R^2^) value of all the response variables were found to be higher and also lack of fit was found to be non-significant (Table 1). The model terms were used to generate response curves for each response variable and the polynomial equations for responses were calculated as significant regression coefficient (β) values-

2 $$\begin{array}{rcl}{{\rm{Y}}}_{{\rm{BERBERINE}}} & = & 17.17-0{{\rm{.56X}}}_{1}+6{{\rm{.20X}}}_{2}\,-\,2{{\rm{.46X}}}_{3}+6{{\rm{.19X}}}_{4}\,-\,1{{{\rm{.99X}}}_{1}}^{2}+3{{{\rm{.07X}}}_{2}}^{2}\\  &  & +\,3{{{\rm{.62X}}}_{4}}^{2}+3{{\rm{.25X}}}_{1}{{\rm{X}}}_{2}\,-\,2{{\rm{.64X}}}_{1}{{\rm{X}}}_{3}\,-\,1{{\rm{.02X}}}_{1}{{\rm{X}}}_{4}\,-\,2{{\rm{.32X}}}_{2}{{\rm{X}}}_{3}+4{{\rm{.32X}}}_{2}{{\rm{X}}}_{4}\end{array}$$

3 $${{\rm{Y}}}_{{\rm{PALMATINE}}}=8.45+3{{\rm{.26X}}}_{2}-1{{\rm{.06X}}}_{3}+2{{\rm{.16X}}}_{4}+1{{\rm{.19X}}}_{1}{{\rm{X}}}_{2}+1{{\rm{.35X}}}_{2}{{\rm{X}}}_{4}$$

4 $${{\rm{Y}}}_{{\rm{TP}}}=12.74+1{{\rm{.79X}}}_{2}+3{{{\rm{.24X}}}_{1}}^{2}-1{{\rm{.31X}}}_{1}{{\rm{X}}}_{3}+0{{\rm{.90X}}}_{1}{{\rm{X}}}_{4}+1{{\rm{.38X}}}_{2}{{\rm{X}}}_{4}$$

5 $$\begin{array}{rcl}{{\rm{Y}}}_{{\rm{ABTS}}} & = & 11.83+1{{\rm{.94X}}}_{1}+0{{\rm{.99X}}}_{2}+2{{\rm{.52X}}}_{4}+2{{{\rm{.16X}}}_{1}}^{2}-1{{{\rm{.80X}}}_{3}}^{2}\\  &  & +\,0{{\rm{.93X}}}_{1}{{\rm{X}}}_{2}-0{{\rm{.72X}}}_{1}{{\rm{X}}}_{3}+0{{\rm{.68X}}}_{1}{{\rm{X}}}_{4}-1{{\rm{.81X}}}_{2}{{\rm{X}}}_{4}\end{array}$$

6 $$\begin{array}{rcl}{{\rm{Y}}}_{{\rm{FRAP}}} & = & 134.83+22{{\rm{.07X}}}_{1}-7{{\rm{.98X}}}_{3}+29{{\rm{.50X}}}_{4}+11{{{\rm{.79X}}}_{1}}^{2}-10{{{\rm{.48X}}}_{2}}^{2}\\  &  & +\,22{{{\rm{.32X}}}_{3}}^{2}-5{{{\rm{.03X}}}_{4}}^{2}-9{{\rm{.19X}}}_{1}{{\rm{X}}}_{3}+13{{\rm{.71X}}}_{1}{{\rm{X}}}_{4}+6{{\rm{.40X}}}_{2}{{\rm{X}}}_{4}+8{{\rm{.13X}}}_{3}{{\rm{X}}}_{4}\end{array}$$

7 $${{\rm{Y}}}_{{\rm{DPPH}}}=34.51+2{{\rm{.14X}}}_{1}+10{{\rm{.20X}}}_{2}+3{{\rm{.05X}}}_{4}+1{{\rm{.28X}}}_{1}{{\rm{X}}}_{2}+1{{\rm{.88X}}}_{2}{{\rm{X}}}_{4}$$

**Table 1:** Regression coefficient (*β*), coefficient of determination (*R*^2^) and *F*-test value of the central composite design (CCD) model for alkaloids, polyphenolics and antioxidant activities.

	*Alkaloids*		*Polyphenolics*	*Antioxidant activity*		
	Berberine	Palmatine	TP (mg GAE/g dw)	ABTS (mM AAE/g dw)	FRAP (mM AAE/g dw)	DPPH (mM AAE/g dw)
*Intercept*, *β*_0_	17.17	8.45	12.74	11.83	134.83	34.51
*β*_1_	−0.56*	0.26	0.45	1.94***	22.07***	2.14***
*β*_2_	6.20***	3.26***	1.79***	0.99**	−1.35	10.20***
*β*_3_	−2.46***	−1.06*	−0.44	−0.38	−7.98***	0.021
*β*_4_	6.19***	2.16***	0.31	2.52***	29.50***	3.05***
*β*_1_^2^	−1.99*	−0.17	3.24**	2.16**	11.79***	1.41
*β*_2_^2^	3.07***	0.48	−1.03	−1.01	−10.48***	−1.70
*β*_3_^2^	1.19	0.70	0.69	−1.80*	22.32***	−1.17
*β*_4_^2^	3.62***	0.60	−1.43	0.016	−5.03*	−0.57
*β*_12_	3.25***	1.19*	0.61	0.93**	−0.54	1.28**
*β*_13_	−2.64***	−0.64	−1.31**	0.72*	−9.19***	−0.14
*β*_14_	−1.02**	0.12	0.90*	0.68*	13.71***	0.71
*β*_23_	−2.32***	−0.86	−0.32	−0.53	0.32	−0.65
*β*_24_	4.32***	1.35**	1.38**	1.81***	6.40***	1.88***
*β*_34_	−0.29	−0.52	−0.22	−0.087	8.13***	−0.44
*R*^2^	0.99	0.89	0.85	0.93	0.99	0.98
*F value* (model)	140.84***	8.82***	6.24***	16.17***	294.54***	75.88***
*F value* (lack of fit)	4.66	135.87	4.26	4.58	4.65	4.67

*β*_1_ = regression coefficient of microwave power, *β*_2_ = sample to solvent ratio, *β*_3_ = solvent pH, *β*_4_ = solvent concentration, TP = total phenols, ABTS = 2,2′-azino-bis (3-ethylbenzothiazoline-6-sulphonic acid) radical cation inhibition, FRAP = Ferric reducing antioxidant power, DPPH = 2,2-diphenyl-1-picrylhydrazyl radical scavenging ability;AAE = ascorbic acid equivalent; GAE = gallic acid equivalent; dw = dry weight. Level of significance *p < 0.05, **p < 0.01, ***p < 0.001.

#### 3D Response Surface Analysis for Alkaloids and Polyphenolic Antioxidants

**Figure 1:**
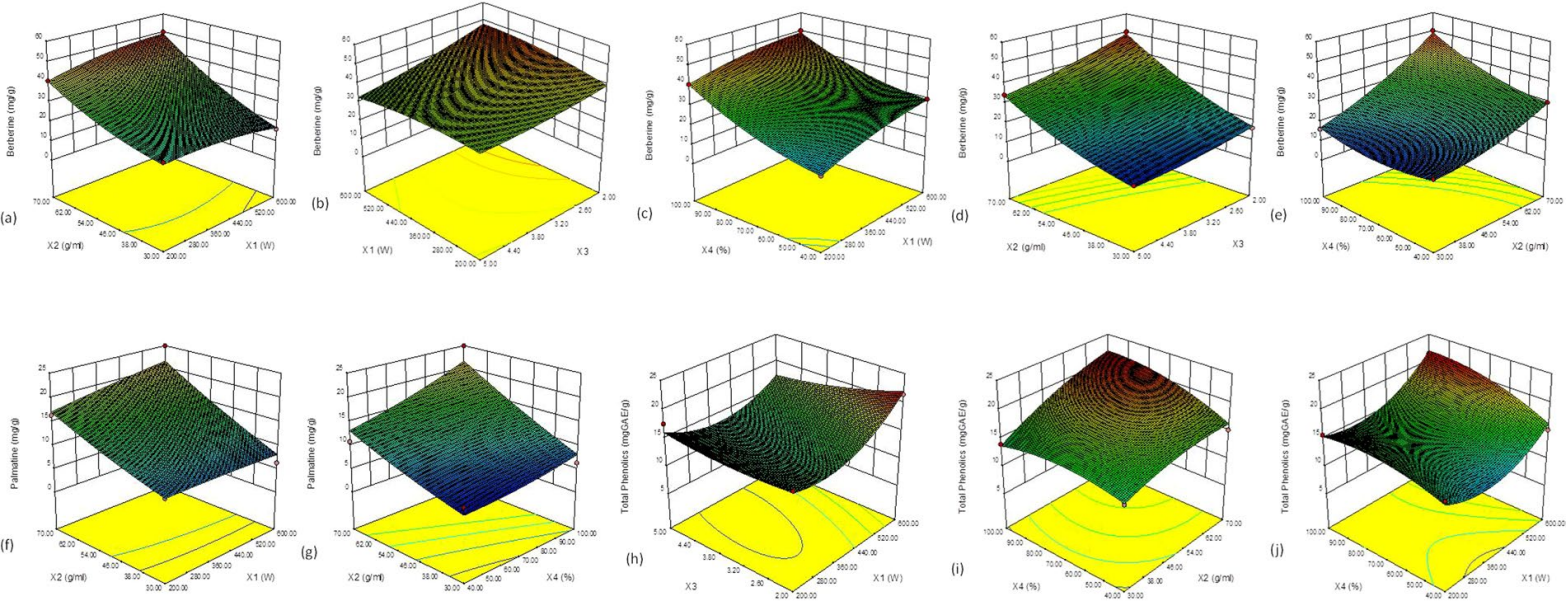
Multiple-component response surface graphs for (**a**–**e**) berberine; (**f**,**g**) palmatine; (**h**–**j**) total phenol (TP) content.

**Figure 2:**
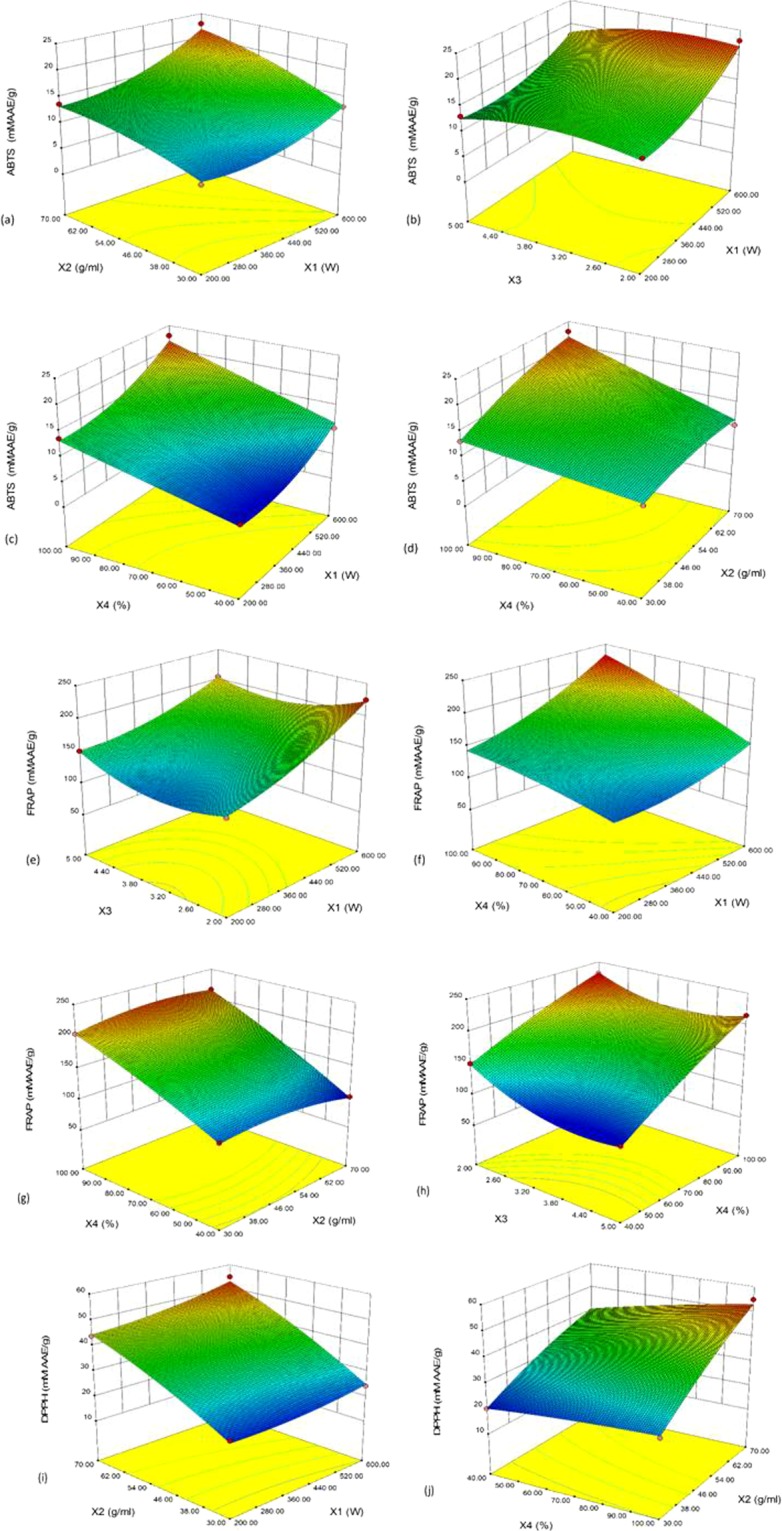
Multiple-component response surface graphs for *in vitro* antioxidant activities (**a**–**d**) ABTS; (**e**–**h**) FRAP; and (**i**,**j**) DPPH.

Effect of microwave power (X1): Microwave power showed significant linear, quadratic and interactive effect on all the responses (Table 1). Berberine concentration decreased significantly (p < 0.05) with increasing microwave power, while a significant (p < 0.001) positive linear effect was found on ABTS, FRAP and DPPH antioxidant activity (Table 1). No significant linear effect of microwave power has been recorded on TP extraction at lower level, however as the power increases above 400 W, a significant positive quadratic effect has been seen (Fig. 1h). For FRAP and ABTS antioxidant activity, a positive significant quadratic effect of microwave power (X_1_) has been recorded at higher levels (Fig. 2a–f).

Effect of sample to solvent ratio (X2): A significant linear positive effect of sample to solvent ratio has been observed for all responses except for FRAP antioxidant activity (Table 1). At higher ratio, above 1:50 gmL^−1^, a significant (p < 0.001) decrease in FRAP activity was recorded as negative quadratic effect (Fig. 2g). However, berberine content was found to be increased with further increasing in solvent volume as a significant (p < 0.001) positive quadratic effect (Fig. 1a,d,e). Interestingly, a significant positive interaction between sample to solvent ratio and solvent concentration (X_1_X_4_) was also recorded for all the responses (Figs. 1 and 2), except for palmatine and DPPH activity. Similarly, a significant positive interactive effect between sample to solvent ratio and microwave power (X_1_X_2_) has been seen for some of the responses. As such, with increasing both X_1_ and X_2_, a significant increase in berberine (p < 0.001) (Fig. 1a), palmatine (p < 0.05) (Fig. 1f), ABTS (p < 0.01) (Fig. 2a) and DPPH antioxidant activity (p < 0.01) (Fig. 2i) have been recorded.

Effect of solvent pH (X3): Significant linear effect of solvent pH on berberine (p < 0.001), palmatine (p < 0.05), and FRAP (p < 0.001) antioxidant activity was recorded (Table 1). With increasing solvent pH, a significant decrease in these responses were recorded (Figs. 1 and 2), Similarly, with increasing solvent pH, FRAP activity decreases at lower methanol concentration (X_4_), however a positive quadratic effect of X_3_ can be seen at higher methanol concentration (X_4_), (Fig. 2h). With increasing solvent pH along with microwave power (X_1_X_3_), a significant negative interactive effect on berberine (p < 0.001) (Fig. 1b), TP (p < 0.01) (Fig. 1h), ABTS (p < 0.05) (Fig. 2b) and FRAP antioxidant activity (p < 0.001) (Fig. 2e) was recorded. Similarly, a significant (p < 0.001) negative interactive effect was found between sample to solvent ratio and solvent pH (X_2_X_3_) on berberine content (Fig. 1d). With increase in ratio along with solvent pH (X_2_X_3_), a significant (p < 0.001) decrease in berberine content was recorded, while lower pH and higher ratio favors berberine extraction (Fig. 1d).

Effect of solvent concentration (X4): For all the responses except TP content, with increasing methanol concentration (X_4_), a significant (p < 0.001) linear increase in response values were recorded, while at higher concentration, a significant (p < 0.05) quadratic negative and significant (p < 0.001) quadratic positive effect was recorded for FRAP antioxidant activity and berberine content, respectively (Table 1, Figs. 1 and 2). With increasing methanol concentration along with microwave power (X_1_X_4_), a significant decrease in berberine content (p < 0.01) was recorded (Fig. 1c), however for TP (p < 0.05) (Fig. 1k), ABTS (p < 0.05) (Fig. 2c) and FRAP antioxidant activity (p < 0.001) (Fig. 2f), an increase in response value was recorded. Interestingly, when both methanol concentration and sample to solvent ratio (X_2_X_4_) acts together, a significant (p < 0.01) increase in all response values have been observed (Figs. 1, 2). However, significant (p < 0.001) positive interactive effect between methanol concentration and solvent pH (X_3_X_4_) was only recorded for FRAP antioxidant activity (Fig. 2h).

### Validation of optimum MAE condition and its comparison

Optimum MAE condition for *B. jaeschkeana* alkaloids and polyphenolics extraction was selected based on the maximum desirability obtained for the model. Under optimum MAE condition, 1 g of root powder sample was mixed with 70 mL of absolute methanol having pH value of 2.0 and the mixture was kept inside the microwave system for 2 min ramp time at 598 W of microwave power. The response values were determined and found very close to the model predicted value, as CV ranges from 0.5 to 5.6% (Table 2). Under these condition, TP (21.27 mg GAE g^−1^), and ABTS (21.96 mM AAE g^−1^), FRAP (230.14 mM AAE g^−1^) and DPPH (53.73 mM AAE g^−1^) antioxidant activities were found to be well fitted with model predicted value (Table 2). Also, berberine and palmatine contents were found 46.38 and 20.54 mg g^−1^, respectively (Table 3, Supplementary Fig. 1a).

**Table 2:** Validation of optimum MAE condition for alkaloids and polyphenolic antioxidants in *B. jaeschkeana* roots and tested the same on *B. asiatica* roots.

Dependent Variables	*Berberis jaeschkeana*			*Berberis asiatica*
	Predicted value	Experimental Value	CV (%)	Experimental value
Berberine (mg/g dw)	49.14	46.38	5.61	88.71
Palmatine (mg/g dw)	21.47	20.54	4.33	18.68
TP (mg GAE/g dw)	21.85	21.27	2.65	30.43
ABTS (mM AAE/g dw)	21.72	21.96	1.09	26.47
FRAP (mM AAE/g dw)	231.30	230.14	0.50	247.37
DPPH (mM AAE/g dw)	52.89	53.73	1.56	66.21

TP = total phenols, ABTS = 2, 2′- azino-bis (3-ethylbenzothiazoline-6-sulphonic acid) radical cation inhibition, FRAP = Ferric reducing antioxidant power, DPPH = 2, 2-diphenyl-1-picrylhydrazyl radical scavenging ability, AAE = ascorbic acid equivalent; GAE = gallic acid equivalent; dw = dry weight, CV = coefficient of variation.

**Table 3:** HPLC-DAD analysis of polyphenolics and alkaloids from *B. jaeschkeana* and *B. asiatica* root under optimum MAE condition.

	*B. jaeschkeana*			*B. asiatica*
Polyphenolic compounds	Retention time (min)		Concentration (mg 100 g^−1^)	Concentration (mg 100 g^−1^)
Gallic acid	3.3		15.0	143.0
(+)-Catechin	3.7		68.0	154.0
Chlorogenic acid	4.2		77.0	276.0
Vanillic acid	5.7		8.0	17.0
Caffeic acid	6.4		nd	6.0
3-hydroxy benzoic acid	6.8		163.0	147.0
Rutin	15.9		nd	466.0
Phloridzin	18.8		4.0	nd
**Alkaloids**				
Berberine	10.7		4638.0	8871.0
Palmatine	5.7		2054.0	1868.0

nd = not detected.

Under optimum MAE conditions, *Berberis asiatica* root samples were extracted and recorded 88.71 mg g^−1^ of berberine and 18.68 mg g^−1^ of palmatine concentration along with TP (30.43 mg GAE g^−1^), and ABTS (26.47 mM AAE g^−1^), FRAP (247.37 mM AAE g^−1^) and DPPH (66.21 mM AAE g^−1^) antioxidant activity (Table 2). Moreover, these plant extracts under optimum MAE conditions showed significantly higher berberine and palmatine contents, as compared to earlier reports (Table 4). The increased yield at lesser extraction time, coupled with simple and environment friendly MAE optimized condition has proven to be an effective green extraction method for these compounds.

**Table 4:** Berberine content of *B. jaeschkeana* and *B. asiatica* species reported from Indian Himalayan Region (IHR).

Species	Extraction method	Berberine concentration (% w/w)	References
*B. asiatica*	0.25 g of root sample extracted with methanol using mortar and pestle.	3.2	^45^
*B. asiatica*	1 g powder root sample was refluxed for 5 min in water bath with 5 mL methanol three times and concentrate under vacuum and final volume make up-to 20 mL.	4.3	^46^
*B. asiatica*	0.25 g of root sample mixed with 20 mL methanol and extracted under microwave reaction system with ramp time = 10 min and hold time of 20 min, IR = 180 °C, Temperature = 80 °C.	1.7–7.7	^28,53^
*B. jaeschkeana*	0.25 g of root sample mixed with 20 mL methanol and extracted under microwave reaction system with ramp time = 10 min and hold time of 20 min, IR = 180 °C, Temperature 80 °C.	1.9–2.9	^47,53^

### HPLC-DAD analysis of polyphenolics under optimum MAE condition

**Figure 3:**
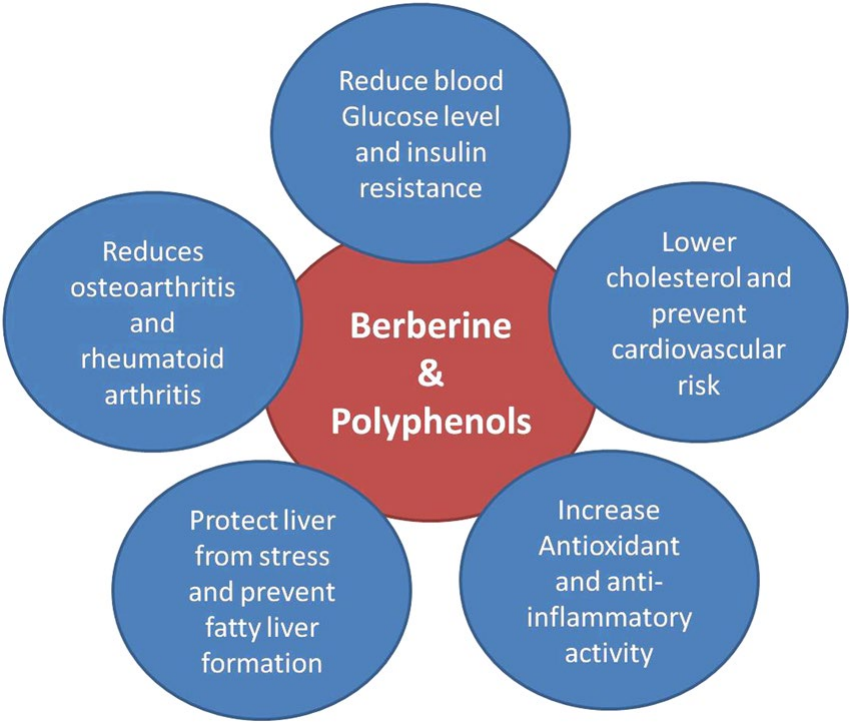
Major pharmacological effects of berberine and polyphenols.

Out of fifteen screened polyphenolic compounds, the HPLC-DAD analysis detected and quantified a total of six polyphenolic compounds in *B. jaeschkeana* and seven in *B. asiatica* roots under optimal MAE condition (Table 3). This study first time detected the presence of phloridzin, 3-hydroxybenzoic acid, chlorogenic acid, vanillic acid, catechin, gallic acid, caffeic acid and rutin in root part of *Berberis* species. The HPLC chromatograms of polyphenolics obtained from *B. Jaeschkeana* roots were presented in Supplementary Figure 1b. In comparison to *B. jaeschkeana*, *B. asiatica* contain polyphenolics in higher concentrations, except for 3-hydroxybenzoic acid. Also, phloridzin was not detected in *B. asiatica*, while caffeic acid and rutin could not detected in *B. jaeschkeana* (Table 3). These polyphenolics are well studied for their nutraceutical properties and found to play important role in combating against various disease conditions^24,25,27,52^. Moreover, these compounds along with berberine were found effective to treat a number of medical complications (Fig. 3).

## Conclusions

An improved microwave-assisted extraction method for pharmaceutically and nutraceutically important natural compounds from *Berberis* species has been developed and validated. Under optimum MAE condition, alkaloids viz. berberine (46.38 mg g^−1^) and palmatine (20.54 mg g^−1^) from *B. jaeschkeana* roots were found higher as compared to earlier reported concentrations. Similarly, optimum MAE condition was also found suitable for extraction of these compounds from *Berberis asiatica* roots, and obtained comparatively higher concentrations to the earlier reports. For the first time six polyphenolic compounds in *B. jaeschkeana* and seven in *B. asiatica* root part have been detected and quantified at optimal MAE condition. Comparison between the species reveals a higher concentration of berberine and polyphenolic antioxidant compounds in *B. asiatica* as compared to *B. jaeschkeana*. As berberine, palmatine and polyphenolic compounds are being commercially used in large number of pharmaceutical and nutraceutical products, the present study provides reliable, repeatable and economical MAE method, which showed improved extraction yield. Also, the multi-component analysis was found to be successful in designing MAE method for extracting valuable compounds of nutraceutical and pharmaceutical importance. Thus, the present study could be replicated in other high-value medicinal plants for their effective and sustainable utilization in nutraceuticals and pharmaceuticals.

## Supplementary information

Supplementary information.
