# Supplementary material for: Optimized microwave assisted extraction (MAE) of alkaloids and polyphenols from Berberis roots using multiple-component analysis
Source: Sci Rep. 2020 Jan 22;10:917. doi: 10.1038/s41598-020-57585-8 (PMC6976575; doi:10.1038/s41598-020-57585-8)
Supplement: Supplementary file 1 — Supplementary information. [file 41598_2020_57585_MOESM1_ESM.pdf]

Optimized microwave assisted extraction (MAE) of alkaloids and polyphenols from Berberis roots using multiple-component analysis

Tarun Belwal[1*], Aseesh Pandey[1, 2 *], Indra D Bhatt[1*], Ranbeer S Rawal[1]

[1]G.B. Pant National Institute of Himalayan Environment and Sustainable Development, Kosi Katarmal, Almora- 263643, Uttarakhand, India

[2]G.B. Pant National Institute of Himalayan Environment and Sustainable Development, Sikkim Regional Centre, Pangthang, Gangtok-737101, Sikkim, India

*Corresponding author. E-mail addresses: draseeshpandey@gmail.com (A.P), tarungbpihed@gmail.com (T.B), bhattid4@gmail.com (I.D.B)

Supplemetary Table 1: Plackett-Burman design (PBD) for estimation of berberine, palmatine and total phenols (TP) as responses under MAE.

	Factors					Responses		
Run	X_1	X_2	X_3	X_4	X_5	Berberine (mg g^{-1})	Palmatine (mg g^{-1})	TP (mg GAE g^{-1})
1	300 (+1)	5 (+1)	50 (+1)	2.5 (-1)	20 (-1)	18.82	8.99	9.94
2	300 (+1)	2 (-1)	50 (+1)	6.0 (+1)	20 (-1)	7.89	7.18	8.78
3	300 (+1)	2 (-1)	20 (-1)	2.5 (-1)	80 (+1)	35.82	7.37	5.91
4	300 (+1)	5 (+1)	20 (-1)	2.5 (-1)	20 (-1)	16.28	4.99	4.73
5	100 (-1)	5 (+1)	50 (+1)	6.0 (+1)	20 (-1)	5.31	6.60	7.47
6	100 (-1)	2 (-1)	20 (-1)	6.0 (+1)	20 (-1)	7.23	3.66	3.92
7	300 (+1)	2 (-1)	50 (+1)	6.0 (+1)	80 (+1)	21.75	9.01	8.67
8	300 (+1)	5 (+1)	20 (-1)	6.0 (+1)	80 (+1)	20.09	5.29	4.58
9	100 (-1)	2 (-1)	50 (+1)	2.5 (-1)	80 (+1)	25.14	9.64	8.90
10	100 (-1)	2 (-1)	20 (-1)	2.5 (-1)	20 (-1)	13.54	4.48	3.97
11	100 (-1)	5 (+1)	20 (-1)	6.0 (+1)	80 (+1)	17.53	5.02	3.99
12	100 (-1)	5 (+1)	50 (+1)	2.5 (-1)	80 (+1)	18.89	8.53	7.28
$\beta_{Berberine}$	2.75*	- 1.21	- 1.06	- 4.06**	5.85***			
$\beta_{Palmatine}$	0.41*	- 0.16	1.59***	- 0.60**	0.75**			
β_{TP}	0.59*	- 0.18	2.00***	- 0.28	0.04			
Model								
F- value						15.48**	41.78***	27.77***
R^2						0.92	0.97	0.95

X_1 = microwave power (W), X_2 = irradiation time (min), X_3 = sample to solvent ratio (g/ml), X_4 =solvent pH, X_5 = solvent concentration (%), TP = total phenols, β = regression coefficient, R^2 = coefficient of determination; Level of significance * $p<0.05$, ** $p<0.01$, *** $p<0.001$

Supplementary Table 2: Central Composite Design (CCD) model with responses as alkaloids, polyphenolics and antioxidant activities for different levels of independent variables.

Run	Independent variables						Dependent variables (Response)			
	X_1	X_2	X_3	X_4	Berberine (mg g^{-1})	Palmatine (mg g^{-1})	TP	DPPH	ABTS	FRAP
1	200 (-1)	30 (-1)	5.0 (+1)	100 (+1)	25	10	11	23	11	151
2	600 (+1)	50 (0)	3.5 (0)	70 (0)	14	8	18	39	17	170
3	600 (+1)	70 (+1)	2.0 (-1)	40 (-1)	30	11	15	40	10	150
4	600 (+1)	30 (-1)	5.0 (+1)	40 (-1)	9	5	10	23	9	116
5	400 (0)	50 (0)	2.0 (-1)	70 (0)	20	9	16	33	10	170
6	400 (0)	50 (0)	3.5 (0)	100 (+1)	26	10	11	35	13	159
7	600 (+1)	30 (-1)	5.0 (+1)	100 (+1)	12	5	11	24	12	204
8	600 (+1)	70 (+1)	2.0 (-1)	100 (+1)	50	25	21	55	23	230
9	600 (+1)	30 (-1)	2.0 (-1)	100 (+1)	16	6	14	24	13	225
10	400 (0)	50 (0)	3.5 (0)	70 (0)	17	9	13	35	12	135
11	400 (0)	50 (0)	5.0 (+1)	70 (0)	17	9	12	33	10	144
12	600 (+1)	30 (-1)	2.0 (-1)	40 (-1)	15	7	13	20	10	162
13	600 (+1)	70 (+1)	5.0 (+1)	40 (-1)	15	10	14	40	9	100
14	400 (0)	50 (0)	3.5 (0)	70 (0)	17	8	12	35	13	136
15	600 (+1)	70 (+1)	5.0 (+1)	100 (+1)	34	15	16	51	18	215
16	400 (0)	30 (-1)	3.5 (0)	70 (0)	15	7	10	24	11	128
17	400 (0)	50 (0)	3.5 (0)	70 (0)	18	9	12	35	13	134
18	200 (-1)	70 (+1)	5.0 (+1)	40 (-1)	13	9	13	36	6	102
19	400 (0)	50 (0)	3.5 (0)	70 (0)	18	9	14	36	12	134
20	200 (-1)	70 (+1)	5.0 (+1)	100 (+1)	36	12	18	43	13	165
21	200 (-1)	30 (-1)	2.0 (-1)	40 (-1)	14	6	13	19	9	131
22	200 (-1)	70 (+1)	2.0 (-1)	40 (-1)	17	10	14	36	6	114
23	400 (0)	50 (0)	3.5 (0)	70 (0)	17	8	13	34	12	137
24	400 (0)	50 (0)	3.5 (0)	70 (0)	17	8	13	36	11	133
25	200 (-1)	30 (-1)	5.0 (+1)	40 (-1)	20	5	18	21	9	116
26	200 (-1)	70 (+1)	2.0 (-1)	100 (+1)	41	16	16	44	14	144
27	400 (0)	70 (+1)	3.5 (0)	70 (0)	25	11	14	41	11	120
28	200 (-1)	50 (0)	3.5 (0)	70 (0)	17	8	14	32	10	123
29	400 (0)	50 (0)	3.5 (0)	40 (-1)	16	8	12	32	10	101
30	200 (-1)	30 (-1)	2.0 (-1)	100 (+1)	22	8	9	22	8	130

X_1 = microwave power (W), X_2 = sample to solvent ratio (g ml^{-1}), X_3 = solvent pH , X_4 = solvent concentration (%), TP = total phenols (mg GAE g^{-1}), DPPH = 2,2-diphenyl-1-picrylhydrazyl radical scavenging ability (mM AAE g^{-1}), ABTS = 2,2'- azino-bis (3-ethylbenzothiazoline-6-sulphonic acid) radical cation inhibition (mM AAE g^{-1}), FRAP = Ferric reducing antioxidant power (mM AAE g^{-1})

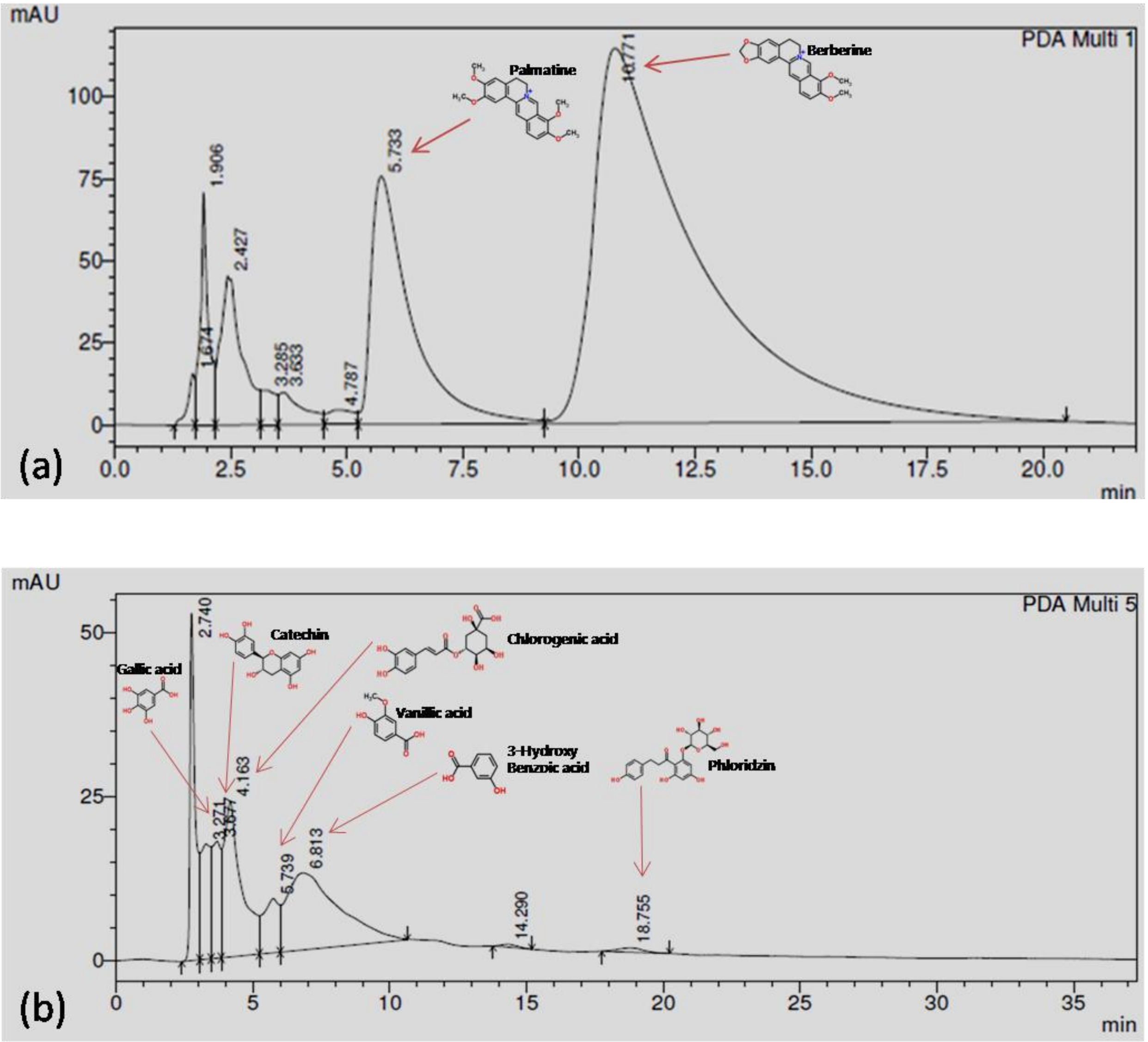

Supplementary Figure 1: Chromatograms of alkaloids and polyphenolic antioxidants in Berberis roots
